# *In vitro* and *in vivo* activities of ticarcillin-loaded nanoliposomes with different surface charges against *Pseudomonas aeruginosa* (ATCC 29248)

**DOI:** 10.1186/2008-2231-20-41

**Published:** 2012-10-03

**Authors:** Amir Gharib, Zohreh Faezizadeh, Masoud Godarzee

**Affiliations:** 1 Department of Laboratory Sciences, Borujerd Branch, Islamic Azad University, Borujerd, Iran; 2 Department of Biology, Borujerd Branch, Islamic Azad University, Borujerd, Iran

**Keywords:** Ticarcillin, Nanoliposome, Pseudomonas aeruginosa, Killing rate, Surface charge

## Abstract

**Background:**

*Pseudomonas aeruginosa* exhibits multiple antibiotic resistance mechanisms. Different studies have shown that entrapment of antibiotics into liposomes could increase their anti-*Pseudomonas* activity. The objectives of this study were to prepare ticarcillin loaded-nanoliposomes with variable surface charges and evaluate their *in vitro* and *in vivo* efficacies against *Pseudomonas aeruginosa* (ATCC 29248).

**Methods:**

Ticarcillin-loaded nanoliposomes with positive, negative and neutral surface charges were prepared by extrusion method. Ticarcillin encapsulation efficacies for different formulations were measured by HPLC method. Minimum inhibitory concentration (MIC) of ticarcillin nanoliposomal forms against strain ATCC 29248 were determined by broth dilution method. The killing rate of *Pseudomonas aeruginosa* was exposed to various concentrations of ticarcillin in free and nanoliposomal forms were analyzed. Ultimately, *in vivo* therapeutic efficacy of nanoliposomes in burned mice skin infected with strain ATCC 29248 was investigated.

**Results:**

The encapsulation efficacies for ticarcillin-loaded cationic nanoliposomes were significantly higher (76% ± 0.17) than those of neutral (55% ± 0.14) and anionic (43% ± 0.14) nanoliposomes. The MIC of free, cationic, neutral and anionic nanoliposomal forms of ticarcillin against ATCC 29248 were to 24, 3, 6 and 48 mg/L, respectively. The killing rates of ticarcillin-loaded cationic nanoliposomes were higher than those of free and other drug formulations. Treatment by ticarcillin-loaded nanoliposomes with positive, neutral and negative surface charges resulted in almost 100, 60 and 20% survival rates, respectively.

**Conclusion:**

Our data suggested that cationic ticarcillin-loaded nanoliposomes because of high effectiveness would be a good choice to treatment of *Pseudomonas aeruginosa* infections.

## Background

*Pseudomonas aeruginosa* is an opportunistic pathogen causing severe, acute and chronic nosocomial infections in hospitals, especially burn units [1]. Since *P. aeruginosa* can rapidly disseminate from the burn wound sites into the blood stream the clinical outcome in these patients can lead to sepsis, which is often fatal [2]. The major problem associated with *Pseudomonas* infection is resistance to conventional antibiotics. Therefore, there is the compelling need to develop novel drug delivery to overcome this resistance [3]. Ticarcillin is an antibiotic belonging to the carboxypenicillin subgroup of third-generation penicillins and covers gram-negative bacteria such as *P. aeruginosa*, but nowadays, this drug is susceptible to degradation by *P. aeruginosa* beta-lactamases [4,5]. Like other antibiotics, increasing time and usage have led to resistance to ticarcillin and so, a delivery system that reducing the ticarcillin-resistant while increasing its therapeutic index is of great interest, and nanoliposomes can provide these benefits [6,7]. Liposomes are spherical and colloidal vesicles that may range from a tens nanometer to several micrometers in size [8,9]. The alterations of liposomal lipids can lead to production of liposomes with variable surface charges [10,11]. Positively or negatively charged liposomes were obtained using both phosphatidylcholine and cholesterol, in combination with stearylamine or dicetylphosphate, respectively [12,13]. Later studies demonstrated that encapsulation of antibiotics into liposomal formulations with different surface charges markedly alters their pharmacokinetics, increasing half-lives and effectiveness [14,15]. Variable results were reported on the *in vitro* and *in vivo* antibacterial activity of liposomal antibiotics against bacteria [7,9]. While some antibiotics such as amikacin are only slowly released from the liposomal carrier, other drugs including ciprofloxacin leak out rapidly [15]. According to the literature, manipulation of physicochemical characteristics of liposomes, like particle size, surface charge, sensitivity to pH changes and bilayer rigidity can have marked effects on the *in vitro* and *in vivo* behavior of antibiotics loaded liposomes and therefore have a major impact on therapeutic success [1,7,14]. The effectiveness of ticarcillin-loaded in nanoliposomes with deferent surface charges yet was not studied. The primary objective of this study was to prepare the ticarcillin-loaded nanoliposomes with three different (neutral, negative and positive) surface charges and evaluate *in vitro* antibacterial activity of their against *P. aeruginosa* (ATCC 29248). A secondary objective was to investigate the therapeutic efficacy of prepared nanoliposomes using a mouse burn model.

## Methods

### Materials

Ticarcillin, stearylamine, dicetylphosphate, cholesterol and egg lecithin was purchased from Sigma Chemical Company (St. Louis, USA) and chloroform, methanol, ammonium acetate and Muller-Hinton broth was purchased from Merck (Darmstadt, Germany).

### Microorganism

*P. aeruginosa* (ATCC 29248) was purchased from American Type Culture Collection (Rockville, MD, USA). For experimentation, this strain was inoculated onto blood agar plates and incubated for 24 h at 37°C.

### Preparation of nanoliposomes

Nanoliposomes were prepared by extrusion method as previously described [16]. Briefly, egg lecithin and cholesterol in the molar ratio of 4:1 were dissolved in chloroform and dried to a lipid film with a rotary evaporator (Brinkman, Toronto, Canada) under N_2_ flow and vacuum at 30°C. The dried lipids were dispersed by agitation in 6 ml of an aqueous solution of ticarcillin (10 mg/ml in PBS, pH 7.4) and sonicated at 4°C in ultrasonic bath (Braun-sonic 2000, Burlingame, USA). At finally, ticarcillin-loaded neutral nanoliposomes were obtained by extruding of respective suspension through a polycarbonate membrane with 100 nm-sized pores for 12 times and separating excess free drug and larger lipid aggregation by centrifugation (100000 g for 30 min). Cationic and anionic ticarcillin-loaded nanoliposomes were prepared and optimized by added stearylamine and dicetylphosphate in nanoliposomal membrane formulations in molar ratios of 0.5, 1, 2 and 3, respectively. Control nanoliposomes were prepared similarly, but PBS (pH 7.4) was used instead of the ticarcillin solution.

### Determination of encapsulation efficacy

The content of ticarcillin in prepared nanoliposomes was determined by HPLC as described previously [17]. Then, the percentage of drug loading was calculated as:

Amount of ticarcillin in nanoliposomes×Total volume tested×100/Total sample volume×Initial amount of ticarcillin

### Particle size, zeta-potential and polydispersity index determination

Mean particle size, zeta-potential and polydispersity index of nanoliposomes was evaluated by the reported method using Malvern zetasizer (Malvern instrument, Worcestershire, UK) apparatus [11].

### Antimicrobial susceptibility testing

The MICs of free and variable ticarcillin-loaded nanoliposomes for *P. aeruginosa* (ATCC 29248) were determined by the broth dilution technique as recommended by CLSI (formerly NCCLS) [18]. Bacterial cell suspensions of ~ 5×10^5^ cells/ml were diluted in Muller-Hinton broth and dispensed (100 μl) into a microtiter tray containing serial two-fold dilutions of ticarcillin. The tray was then incubated for 24 h at 37°C. The MIC was recorded to be the lowest concentrations of ticarcillin in free and nanoliposomal forms that prevented visible bacterial growth and expressed in μg/ml.

### Time-kill studies

Time kill studies were preformed in triplicate in 10 ml tubes containing 2 ml of Mueller-Hinton broth as described previously [19]. In brief, 100 μl of *P. aeruginosa* suspension were resuspended in 10 ml of Mueller-Hinton broth and incubated overnight at 37°C, and adjusted to a McFarland standard of 0.5. Then, 100 μl of this standardized inoculum were added to separate culture tubes containing 1 ml of Mueller-Hinton broth with 1 ml ticarcillin solutions in free and different nanoliposomal forms at 1, 2 and 4 times the MIC and incubated at 37°C. At finally, colony counts were performed onto Trypticase soy agar (TSA) plates at 0, 2, 4, 6 and 24 h and the results were expressed as log colony forming unit (CFU)/ml.

### *In vivo* study

*In vivo* therapeutic efficacies of ticarcillin-loaded nanoliposomes were tested by a described method [20], with some modification. In brief, sixty male BALB/c mice (20–22 g) obtained from the National Institute of Pasture, Iran. Animals were handled according to the national guidelines of the laboratory animal and housed in separate and pathogen- free cages and received food and water *ad libitum*[21]. All groups were anesthetized with ketamine-xylazine mixture (50 mg/kg each, given intramuscularly), and their backs were shaved. To induce burn in the backs of mice, a brass bar (10 by 10 by 100 mm) was heated in boiling water for 15 min and applied on the shaved back of the animals for 45 s. Then, 50 μl of the bacterial inoculum (containing 10^9^ CFU of total bacteria) was applied subcutaneously into the sites of the burn on the animal’s back. The mice were divided into 5 groups. All groups were treated topically as follows: Group 1 received cationic ticarcillin-loaded nanoliposomes (75 mg/kg/12h); group 2 received neutral ticarcillin-loaded nanoliposomes (75 mg/kg/12h); group 3 received anionic ticarcillin-loaded nanoliposomes (75 mg/kg/12h); group 4 received anionic ticarcillin-loaded nanoliposomes (75 mg/kg/12h); group 5 received empty nanoliposomes (75 mg/kg/12h), and group 6 received physiological saline (1 ml/kg/12h); for 7 days starting from the 3rd day post infection. Two days after the last dose the surviving animals were anesthetized and sacrificed by cervical dislocation and the liver, kidney, spleen and skin of mice were removed under sterile conditions and homogenized for 5–10 min in PBS (2 ml/g). The homogenates were serially diluted and plated for growth in TSA. The inoculated plates were then incubated at 37°C for 24 h and the colony forming unit (CFU) was counted.

### Data analysis

The results were expressed as means ± standard errors of means. The data of killing rate study were statistically evaluated by paired Student’s *t*-test, and *P value* of less than 0.05 was considered significant. The survival rates of control and treated mice were determined by using chi-squared with Yates correction and by Fisher’s exact test. Therefore, these data were used to prepare of cationic and anionic ticarcillin-loaded nanoliposomes.

## Results

### Encapsulation efficacy

The results showed that encapsulation efficacies of ticarcillin in cationic, neutral and anionic nanoliposomal forms were 76% ± 0.17, 55% ± 0.21 and 43% ± 0.14, respectively. Tables 1 and 2 shows the highest encapsulation efficacies of ticarcillin into cationic and anionic nanoliposomes were obtained by the addition of 1 molar of stearylamine and dicetylphosphate to formulations, respectively.

**Table 1:** Influence of stearylamine molarity in encapsulation efficacy*

	**Molarity of stearylamine**			
	**0.5**	**1**	**2**	**3**
**Encapsulation efficacy**	70 ± 0.14	76% ± 0.17**	67 ± 0.32	63 ± 0.18

* The Data are expressed as Mean ± S.E.M.

** Significant difference (*p* < 0.05).

**Table 2:** Influence of dicetylphosphate molarity in encapsulation efficacy*

	**Molarity of dicetylphosphate**			
	**0.5**	**1**	**2**	**3**
**Encapsulation efficacy**	38 ± 0.34	43% ± 0.14**	34 ± 0.11	27 ± 0.30

* The Data are expressed as Mean ±S.E.M.

** Significant difference (*p* < 0.05).

### Particle size, zeta-potential and polydispersity index analysis

Table 3 shows the mean particle size, zeta-potential and polydispersity index of empty and ticarcillin loaded-nanoliposomes with variable surface charges. Size homogeneity of empty and loaded-nanoliposomes suggested that ticarcillin was entrapped into lipid bilayer, according to the previous studies [14,21]. Zeta-potential of nanoliposomes revealed that prepared nanoparticles have appropriate stability in aqueous dispersion [22]. 

**Table 3:** Particle size, zeta-potential and polydispersity index of empty and ticarcillin-loaded nanoliposomes

**Formulations**	**Mean particle size (nm)**	**Zeta-potential (mV)**	**Polydispersity index**
**Empty**			
Neutral liposomes	94.9 ± 0.15	−1.1 ± 0.27	0.22 ± 0.01
Anionic liposomes	93.2 ± 0.36	−15.7 ± 0.95	0.23 ± 0.04
Cationic liposomes	95.3 ± 0.49	+20.1 ± 0.52	0.25 ± 0.02
**Loaded**			
Neutral liposomes	95.2 ± 0.31	−1.1 ± 0.27	0.23 ± 0.02
Anionic liposomes	92.6 ± 0.44	−15.2 ± 0.86	0.26 ± 0.01
Cationic liposomes	96.1 ± 0.14	+21.8 ± 0.13	0.25 ± 0.03

### Bacterial susceptibilities

The MICs values ticarcillin in either free or nanoliposomal forms for *P.aeruginosa* (ATCC 29248) was shown in Table 4. The MICs of free, cationic and neutral nanoliposomal forms of ticarcillin were higher than anionic formulation, respectively.

**Table 4:** ***In vitro *antimicrobial activities of free and nanoliposomal forms of ticarcillin for *P. aeruginosa *(ATCC 29248)**

**Minimum inhibitory concentration (mg/L)**				
**Drugs**	**Free ticarcillin**	**Tticarcillin-loaded nanoliposomes**		
		**Cationic**	**Neutral**	**Anionic**
Microorganism				
***P. aeruginosa *(ATCC 29248)**	24	3	6	48

### Time-killing study

**Figure 1:**
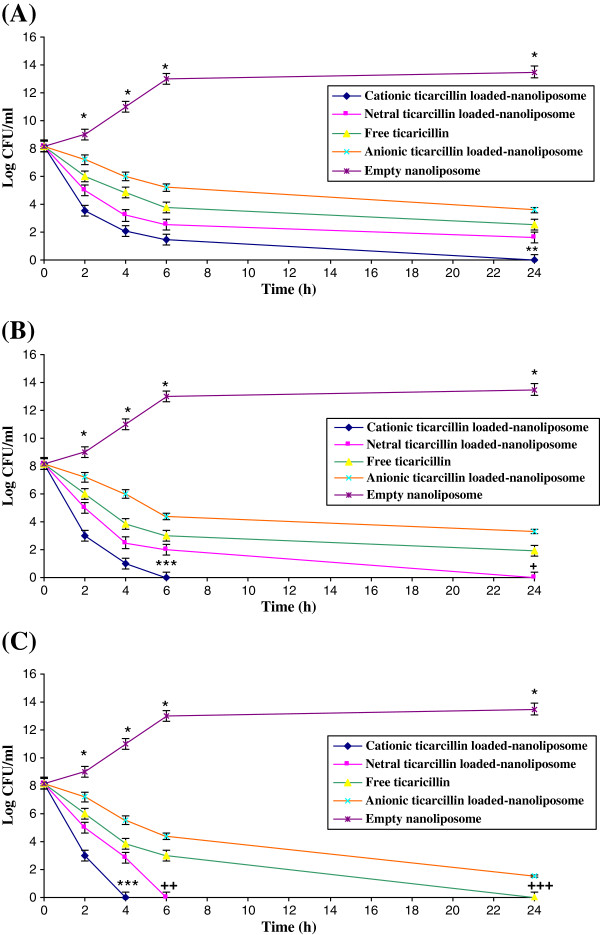
**Killing curves for *P. aeruginosa *(ATCC 29248) was exposed to various concentrations (A=1×MIC, B=2×MIC and C=4×MIC) of ticarcillin in free and neutral, negative and positive nanoliposomal forms.** *Significant difference between killing rate of empty nanoliposomes versus free and another loaded nanoliposomes (p<0.01), **Significant difference between killing rate of cationic nanoliposomes versus free and another loaded nanoliposomes (p<0.05), ***Significant difference between killing rate of cationic nanoliposomes versus free and another loaded nanoliposomes (p<0.01), ^**+**^Significant difference between killing rate of neutral nanoliposomes versus free and anionic nanoliposomes (p<0.05), ^**++**^Significant difference between killing rate of free drug versus anionic nanoliposomes (p<0.05). ^**+++**^Significant difference between killing rate of cationic nanoliposomes versus free and another loaded nanoliposomes (p<0.01).

The killing curves of ticarcillin in free and encapsulated forms at 1, 2 and 4 times the MICs was shown in Figure 1. In all conditions free ticarcillin was more effective on reduced bacterial counts as compared with ticarcillin encapsulated in anionic nanoliposomes (Figure 1). At one times of MIC only ticarcillin encapsulated in cationic nanoliposomes could eliminate of *P. aeruginosa* after 24 h (Figure 1-A). At two times of MIC both ticarcillin encapsulated in cationic and neutral nanoliposomes eradicated the bacteria after 6 and 24 h, respectively (Figure 1-B). At four times of MIC, both ticarcillin in free and encapsulated in cationic and neutral nanoliposomes could eliminate the *P.aeruginosa* (ATCC 29248) after 4, 8 and 24 h, respectively (Figure 1-C).

### Therapeutic efficacy test

The treatment of the *P. aeruginosa*-infected burned mice with ticarcillin-loaded cationic nanoliposomes compared to control animals showed significant reduction in CFU values in evaluated organs, especially in skin, spleen and liver (Table 5). It was found that mortality of mice as control (without AmB administrated) was 100% after 7 days, whereas mice treated with ticarcillin in free and encapsulated in cationic, neutral and anionic nanoliposomes showed the increase in survival rate of 30, 100, 60 and 20%, respectively.

**Table 5:** **The survival rate of infected burned mice and colony-forming units (CFUs) of *P. aeruginosa *(ATCC 29248) in different organs**

**Treatment**	**Tissue/Organ**	**Log CFU/ Gram tissue (n=3)**	**Percentage of survival mice (n=10)**
Control without drug administration (received physiological saline, Topically)	Liver	3.211 ± 0.5	None survived
	Kidney	3.750 ± 0.3	
	Spleen	3.829 ± 0.6	
	Skin	3.690 ± 0.2	
Empty nanoliposomes (1 mg Kg^-1^, Topically)	Liver	3.355 ± 0.4	None survived
	Kidney	3.960 ± 0.8	
	Spleen	3.764 ± 1.5	
	Skin	3.590 ± 1.3	
Free ticarcillin (1 mg Kg^-1^, Topically)	Liver	2.315 ± 0.04	30
	Kidney	2.198 ± 0.07	
	Spleen	2.312 ± 1.3	
	Skin	2.420 ± 0.04	
Anionic ticarcillin-loaded nanoliposome (1 mg Kg^-1^, Topically)	Liver	2.715 ± 0.8	20
	Kidney	2.910 ± 0.2	
	Spleen	2.650 ± 1.4	
	Skin	2.680 ± 1.1	
Neutral ticarcillin-loaded nanoliposome (1 mg Kg^-1^, Topically)	Liver	1.712 ± 0.6	60
	Kidney	2.045 ± 0.1	
	Spleen	1.024 ± 1.1	
	Skin	1.362 ± 0.04	
Cationic ticarcillin-loaded nanoliposome (1 mg Kg^-1^, Topically)	Liver	Nil^*^	100
	Kidney	1.013 ± 0.07^**^	
	Spleen	Nil^*^	
	Skin	Nil^*^	

The Data are expressed as Mean±Standard error of mean from three separate experiments. Analysis of variance of one-way classification between the treatment means was heterogeneous and the *t*-test values (two-tailed) were significant, ^*^p <0.001 and ^**^p < 0.05.

## Discussion

The use of liposomes as antibiotic carrier systems has been widely investigated [11,20]. However, the main problems associated with application of liposomes as carriers for antibiotics is insufficient quantities in the target site [1,18]. In addition, the preparation of antibiotic-loaded liposomes with high encapsulation efficacy may not be easy because the variable interactions between antibiotics and bilayer lipids can occur [2,11]. For the elimination of this problem, investigators were changed lipids in liposomal membrane formulation. In this report, we evaluated the potential of incorporation of ticarcillin into cationic, neutral and anionic nanoliposomes. The results showed that ticarcillin can be encapsulated into three kinds of nanoliposomes with variable entrapment efficacy. It has been shown that the highest encapsulation efficiencies occur when lipids and loaded drugs have opposite charges [13,15]. In this case, we previously reported that superoxide dismutase (SOD) with negative surface charge has high encapsulation efficacy in cationic liposomes [12]. Whereas the electric charge of ticarcillin in the pH 7.4 is negative [20], it probably makes the encapsulation efficacy of ticarcillin in cationic nanoliposomes were higher than neutral and anionic nanoliposomes, respectively. Our results suggest that entrapped of ticarcillin in neutral and cationic nanoliposomal forms enhanced the antipseudomonal activity of its compared to free ticarcillin. Our data fits well with some other studies. We previously studied that amikacin-loaded neutral liposomes displayed stronger bactericidal activity than the free drug [9]. Furthermore, it has been reported that encapsulated of cephalexin in liposomes with neutral and negative surface charges could protect the drug from hydrolysis by *staphylococcal* β-lactamase as well [22]. Several hypotheses, including increased electrostatic impulsion and protection of the antibiotics from bacterial enzymes may explain the mechanism of enhanced antimicrobial activities of liposomal formulations [7,14,23]. Additionally, the molecular configuration of antibiotics within liposomes could play an important role in these cases [18]. The time-kill assays confirmed that higher potency of cationic and neutral nanoliposomal forms of ticarcillin than free antibiotics. These results are in accordance with previous findings, which reported that a significantly higher killing rate of *P. aeruginosa* with meropenem and gentamicin-loaded cationic liposomes were occurred [24]. So, we hypothesized that electrostatic interaction between the outer membrane lipopolysaccharides of *P. aeruginosa* and cationic nanoliposome loaded-ticarcillin could enhance the mechanism of drug entry into this microorganism cell. Burns, wounds, and other exposed tissues are particularly susceptible to microbial contamination and infections [25,26]. It is proven that potential mortality from burn wound infections, even with aggressive antibiotic therapy, may approach or exceed 50% [27]. Treatment of mice with ticarcillin-loaded in cationic nanoliposomes resulted in 100% survival rate and in almost complete eradication of the bacteria from the spleens, livers, and skins of infected animals. These results may be due to the optimal antibiotic delivery that reported by several investigators [20,28]. When cationic liposomes containing antibiotics are applied topically; they may interact with the cell membranes of exposed tissues and therefore, protects the burn wound tissues from further bacterial contamination [29].

## Conclusion

*In vitro* and *in vivo* testing of nanoliposomes indicated that ticarcillin-loaded in cationic nanoliposomes have a stronger protective effect against *P. aeruginosa* infection compared to free, neutral and anionic ticarcillin formulations. Therefore, we suggest that effective formulations would be a good choice for treatment of patients with *P. aeruginosa* infections, especially in burn units of hospitals.

## Competing interests

The author(s) declare that they have no competing interests.

## Authors’ contributions

All authors have read and approved the final manuscript.
